# Brain transcriptional stability upon prion protein-encoding gene invalidation in zygotic or adult mouse

**DOI:** 10.1186/1471-2164-11-448

**Published:** 2010-07-22

**Authors:** Sead Chadi, Rachel Young, Sandrine Le Guillou, Gaëlle Tilly, Frédérique Bitton, Marie-Laure Martin-Magniette, Ludivine Soubigou-Taconnat, Sandrine Balzergue, Marthe Vilotte, Coralie Peyre, Bruno Passet, Vincent Béringue, Jean-Pierre Renou, Fabienne Le Provost, Hubert Laude, Jean-Luc Vilotte

**Affiliations:** 1 INRA, UMR1313, Génétique Animale et Biologie Intégrative, F-78350, Jouy-en-Josas, France; 2 UMR INRA 1165 CNRS 8114, Recherche en Génomique Végétale, UEVE, F-91057 Evry Cedex, France; 3 UMR AgroParisTech/INRA Mathématique et Informatique Appliquées 518, F-75005 Paris, France; 4 INRA, UE 907, Unité Expérimentale Animalerie Rongeur, F-78350 Jouy-en-Josas, France; 5 INRA, UR892, Virologie Immunologie Moléculaires, F-78350 Jouy-en-Josas, France

## Abstract

**Background:**

The physiological function of the prion protein remains largely elusive while its key role in prion infection has been expansively documented. To potentially assess this conundrum, we performed a comparative transcriptomic analysis of the brain of wild-type mice with that of transgenic mice invalidated at this locus either at the zygotic or at the adult stages.

**Results:**

Only subtle transcriptomic differences resulting from the *Prnp *knockout could be evidenced, beside *Prnp *itself, in the analyzed adult brains following microarray analysis of 24 109 mouse genes and QPCR assessment of some of the putatively marginally modulated loci. When performed at the adult stage, neuronal *Prnp *disruption appeared to sequentially induce a response to an oxidative stress and a remodeling of the nervous system. However, these events involved only a limited number of genes, expression levels of which were only slightly modified and not always confirmed by RT-qPCR. If not, the qPCR obtained data suggested even less pronounced differences.

**Conclusions:**

These results suggest that the physiological function of PrP is redundant at the adult stage or important for only a small subset of the brain cell population under classical breeding conditions. Following its early reported embryonic developmental regulation, this lack of response could also imply that PrP has a more detrimental role during mouse embryogenesis and that potential transient compensatory mechanisms have to be searched for at the time this locus becomes transcriptionally activated.

## Background

The pivotal role that the prion protein (PrP) plays in transmissible spongiform encephalopathies (TSE) is now well established [[1,2] for recent reviews]. The conversion of this host-encoded protein to an abnormal, partially proteinase K resistant, isoform is a hallmark of most TSEs and PrP is the only known constituent of mammalian prions [3]. The *Prnp *gene that encodes for PrP, is expressed in a broad range of vertebrate tissues but most abundantly in the central nervous system [4].

Although PrP is evolutionary conserved, suggesting that it has an important role, its physiological function remains unclear even though its implication in neuroprotection, response to oxidative stress, cell proliferation and differentiation, synaptic function and signal transduction has been proposed [5,6]. Its temporal regulation led also to suspect an implication of this protein in early embryogenesis [7-9] but *Prnp*-knockout mice [10,11], cattle [12] and goat [13] were obtained with no drastic developmental phenotype and only subtle alterations of their circadian rhythm, hippocampal function and of their behavior. A similar observation was made when this gene was invalidated in adult neurons [14,15]. To explain these data, it was hypothesized that another host-encoded protein is able to compensate for the lack of PrP [16]. However, this protein has not yet been identified.

Transcriptomic analysis has emerged as a powerful tool to decipher cellular pathways that are modified following a gene expression alteration as it does not pre-require the need of restricting hypothesis. Such approaches have been conducted to analyze the mechanisms underlying prion replication and neurotoxicity [see [17-24] for recent examples]. The obtained results appeared however inconsistent and closely related to the cell type and/or strain and animal model used, leading to difficulties in identifying the metabolic pathways involved.

Fewer studies have used a similar approach to try to understand the biological function of the PrP protein in immortalized non-neuronal cells [25-27]. The obtained results appear again to correlate with the cell line used as experimental model and no shared pathway has emerged from the comparison of these different experiments. In parallel, proteomic studies have been conducted either using two cell lines [28] or transgenic knockout mice [29]. While different sets of proteins were found to be affected by the PrP expression level in cells according to their origin, no significant difference was detected in the brain proteome of the analyzed 129/Sv-C57/Bl6 transgenic mice, bearing in mind that variations occurring for low abundant proteins might not have been detected [29].

In the present study, we report the comparisons of the whole brain transcriptomes of PrP knockout or wild type mice, both on an FVB/N genetic background, and of that of mice invalidated for the Prnp locus in adult neurons.

## Results and Discussion

### Comparative transcriptional analysis of FVB/N versus FVB/N Prnp^-/- ^mouse brains

A search for differentially expressed genes was done by comparison of the expression profiles of FVB/N versus FVB/N Prnp-/- [10,30] mouse brains. To this aim, RNA samples were prepared by pooling RNAs from 5 brains of 6-week old mice of each genotype. After statistical analysis, two genes were found to be differentially expressed, including the *Prnp *one (Table 1). The relatively low log ratio observed for the variation in *Prnp *expression is explained by the fact that the knockout experiment was done in such a way that the gene remains expressed although at a lower level, around 2 to 3 fold, as observed by Northern blotting (data not shown), but that the resulting mRNA does not encode for PrP anymore [10].

**Table 1:** Candidate genes resulting from microarray studies comparing FVB/N *Prnp*^-/- ^versus FVB/N mice.

Genes	FVB/N *Prnp*-/-	FVB/N *Prnp*-/-	Protein
	**Microarray**	**QPCR**	
	**(Log2 ratio)**	**(ΔΔ CT)**	

Downregulated			
*Prnp*	-1.63	-15	Prion protein or PrPc
*Scg5*	-1.37	-2.13	Neuroendocrine secretory protein 7B2
Upregulated			
None			

Differentially expressed genes detected by the microarray analysis are listed with the calculated differential ratio (log2) alongside the observed delta-delta CT resulting from the QPCR experiment. The comparative C_t _method is known as the 2 ^[delta][delta]Ct ^method, where delta delta C_t _= [delta]C_t, sample _- [delta]C_t, reference_

QPCR was applied to confirm the suspected differential expression of the detected genes using the same pools of brain RNAs. Both *Prnp *and *Scg5 *differential expression were confirmed (Table 1). The higher relative fold-change observed by QPCR for the *Prnp *gene compared to that detected in the micro-array experiment is related to the location of the used primers in exon 2 and in exon 3 of the PrP-encoding gene respectively, which will not amplify the retro-transcribed RNA expressed by the invalidated locus.

The *Scg5 *encodes the 7B2 neuroendocrine secretory protein, a specific chaperone for the proprotein convertase 2 [31]. Invalidation of this gene leads to a hypersecretion of cortocitropin that induces early lethality. This protein function was of interest in regards with PrP since hypercorticism is a phenotype associated with scrapie in ewes [32]. However, a search in the mouse genome database for the chromosomal localization of the *Prnp *and *Scg5 *loci revealed that these two genes are physically linked and only 11 cM apart. It has been reported that the level of expression of 7B2, at least in the pancreas, differs between mouse strains and is related to a genetic polymorphism that occurs within its proximal promoter [33]. Since the *Prnp *knockout was done on 129/Sv ES cells, we hypothesized that the *Scg5 *gene could still be of a 129/Sv genetic origin in FVB/N *Prnp*^-/- ^mice while it is of FVB/N genetic origin in wild-type mice. We amplified by PCR and sequenced the -200/-60 *Scg5 *promoter region starting from genomic DNA of three FVB/N, 129/Sv and FVB/N *Prnp*^-/- ^mice, respectively. A single nucleotide polymorphism (G/T) could be detected at position -97 that discriminates between the FVB/N and the 129/Sv or FVB/N *Prnp*^-/- ^genotypes (Table 2). These results thus indicate that in the FVB/N *Prnp*^-/- ^mice, the *Scg5 *locus remains of 129/Sv genetic origin. It is worth mentioning that the detected single nucleotide polymorphism abolishes a putative AML 1A transcription factor binding site, TGGGGT, in the FVB/N *Scg5 *promoter possibly explaining the different levels of expression observed. Altogether, these data suggest that the brain *Scg5 *differential expression between the FVB/N and FVB/N *Prnp*^-/- ^mice results from a different genetic origin of this locus.

**Table 2:** Single nucleotide polymorphism observed within the *Scg5 *proximal promoter region.

	...-111	-87....
**FVB/N**	....CAGGGCTTAAGTGC**G**GGGGTAGGAAA	
**FVB/N *Prnp*-/-**	....CAGGGCTTAAGTGC **T**GGGGTAGGAAA	
**129**	....CAGGGCTTAAGTGC **T**GGGGTAGGAAA	

The sequences were obtained from three independent mice of each genotype. The sequences are numbered backward starting from the reported distal-most transcription initiation site [33]. The observed single nucleotide polymorphism is indicated in bold-faced type.

The observed poor transcriptional alteration in the brain of mouse depleted for PrP could suggest that these animals adapted to this genetic environment during embryogenesis. If such, invalidation of the PrP-encoding gene at an adult stage might induce detectable transient modification of the genome transcriptomic regulation. Using already validated conditional knockout transgenic lines [14], we analyzed the potential impact of an adult neuronal PrP depletion on the overall brain transcriptome.

### Incidence of a post-natal neuronal PrP depletion on the brain transcriptome

This experiment was performed using Tg37 mice, transgenic mice expressing physiological levels of mouse PrP from a transgene composed of a the floxed coding sequences inserted within the hamster-based CosShaTet expression vector, crossed with NFH-Cre transgenic mice [14]. Both transgenic mice were under a mouse *Prnp*-knockout genetic background. The brain RNA pools consisted of littermates of 2 males and 2 females of either 10 or 14 weeks old for each Tg37^+/- ^NFH-Cre^-/- ^or Tg37^+/- ^NFH-Cre^+/- ^genotypes. These two ages were chosen since activation of the NFH promoter results in ablation of PrP in neurons after 9 weeks [14]. After statistical analysis, 11 and 47 genes were found to be differentially expressed at 10 (Table 3) or 14 (Table 4) weeks, respectively. At 10 weeks, 3 genes were over-expressed and 8 under-expressed in *Prnp *depleted mice as compared with the NFH-Cre-negative control animals. At 14 weeks, 21 and 26 genes were over-expressed or under-expressed, respectively. Because the oligonucleotide that recognized *Prnp *in the microarray is located within the 3' UTR of the gene, a region poorly conserved, it was not expected to detect expression of the Tg37 transgene that encompasses the hamster *Prnp *3'UTR sequence and indeed differential expression of this gene was not revealed. The absence of the *Scg5 *locus within the detected differentially expressed genes further supports the above hypothesis that explains its detection in the previous experiment by a physical link between the *Scg5 *and the *Prnp *loci rather than by a functional one.

**Table 3:** Differentially expressed genes detected at 10 weeks by microarray studies comparing Tg37^+/^^- ^NFH-Cre^-/^^- ^and Tg37^+/^^- ^NFH-Cre^+/^^- ^brain tissues.

*Microarray ID*	*Locus Name*	*Ratio (log2)*	*Bonf Pval*	Top Functions
301955	**Atp13a1**	0,81	2,90E-09	*Response to oxidative stress*
217673	A630023P12Rik	-0,62	3,16E-04	*Unknown*
250792	*Txnl2*	-0,60	8,65E-04	*Cardiovascular system development, Neuronal differentiation*
262559	*Zfp819*	-0,58	2,89E-03	*Embryonic Development*
203516	**Sepx1**	-0,56	7,29E-03	Genetic Disorder
284620	**Slc8a1**	-0,54	1,74E-02	*Response to oxidative stress*
235326	***Kcnj5***	-0,54	2,31E-02	Cell Death, Neurological Disease, Nervous System Development and Function
202068	**1810030J14Rik**	-0,53	3,99E-02	*Cancer, Cell death*
268919	A130070M06	-0,52	4,95E-02	*Ribosome release*
253726	***Synaptotagmin11***	0,52	4,38E-02	*Synaptic vesicle trafficking, Nervous system function, Response to oxidative Stress*
247145	0610038F07Rik	0,58	3,43E-03	*Mitochondrial function*

Differentially expressed genes detected by the microarray analysis are listed with the calculated differential ratios (log2) and the Bonferroni *p *values (Bonf Pval). The top functions were deduced either using the Ingenuity pathways analysis software http://www.ingenuity.com or by looking at the expression pattern and putative functions attributed to those genes (italized annotations). Italic names: genes potentially involved in cellular development and differentiation. Bold-faced type names: genes potentially involved in cell death and disorders, including response to oxidative stress. Italic and bold-faced type names: genes potentially involved in both sets of functions.

**Table 4:** Differentially expressed genes detected at 14 weeks by microarray studies comparing Tg37^+/^^- ^NFH-Cre^-/^^- ^and Tg37^+/^^- ^NFH-Cre^+/^^- ^brain tissues.

Microarray ID	Locus Name	*Ratio (log2)*	*Bonf Pval*	Top Functions
**275404**	*EST1 (Genebank *AV451297.1*)*	**-2,62**	**0,00E+00**	**Unknown (Embryonic Development)**
**197253**	*Ifitm3*	**-1,41**	**0,00E+00**	**DNA replication, Nervous System Development and Function**
**237827**	AY036118	**-1,19**	**0,00E+00**	*Eukaryotic polypeptide chain releasing factor*
**245680**	4931406E20Rik	**-0,92**	**0,00E+00**	***Unknown***
**202068**	1810030J14Rik	**-0,89**	**0,00E+00**	***Unknown (Cancer, Cell death)***
**196280**	*Prelid2*	**-0,81**	**1,40E-10**	**Cardiovascular Disease, Cellular Development, Embryonic Development**
**211028**	4930428E07Rik	**-0,79**	**6,77E-10**	***Unknown (Reproductive System)***
**272796**	6430604K15Rik	**-0,76**	**7,67E-09**	***Unknown (Zinc Finger protein)***
**312533**	BM229693	**-0,71**	**2,99E-07**	***Unknown (Embryonic Development)***
**231366**	Tmem98	**-0,69**	**7,27E-07**	***Unknown (Transmembrane protein)***
**242062**	*Sdccag3*	**-0,65**	**1,11E-05**	**Cancer, Cardiovascular System Development and Function, Reproductive system Disease**
**226542**	*Ifng*	**-0,62**	**6,35E-05**	**Cardiovascular Disease, Cellular Development, Embryonic Development Cell Death, Neurological Disease, Nervous System**
**281732**	***Papss2***	**-0,60**	**1,62E-04**	**Development and Disease Cell Death, Neurological Disease, Nervous System**
**202885**	***Ramp1***	**-0,58**	**7,58E-04**	**Development and Disease**
**213956**	9930022N03Rik	**-0,58**	**8,07E-04**	***Unknown (expressed in dendtritic cells)***
**253726**	***Synaptotagmin11***	**-0,56**	**1,77E-03**	***Synaptic vesicle trafficking, Nervous system function, Response to oxidative stress***
**218976**	4833414E09Rik	**-0,55**	**3,39E-03**	***Unknown (expressed in skin and neonate head)***
**273728**	*Zbtb33*	**-0,55**	**3,81E-03**	**DNA replication, Nervous System Development and Function**
**277491**	*Adam24*	**-0,54**	**5,33E-03**	**Reproductive System Development and Function**
**300948**	4930579C12Rik	**-0,54**	**5,41E-03**	***Unknown (Reproductive system)***
**207253**	C330013F16Rik	**-0,54**	**7,11E-03**	***Unknown***
**279418**	A530088H08Rik	**-0,53**	**8,21E-03**	***Unknown***
**287029**	*Fryl*	**-0,53**	**9,99E-03**	**Cardiovascular Disease, Cellular Development, Embryonic Development**
**287258**	Grin1	**-0,52**	**1,26E-02**	**Carbohydrate metabolism, Lipid metabolism, Small molecule Biochemistry**
**312507**	9030411K21Rik	**-0,51**	**2,50E-02**	***Unknown (Embryonic Development)***
**192336**	BC043118	**-0,50**	**3,79E-02**	***Unknown (Nervous System Development)***
**217372**	Sec1	**0,50**	**3,28E-02**	***Synaptic transmission and general secretion***
**241944**	2810471M01Rik	**0,51**	**2,15E-02**	***Unknown***
**214574**	**Cabp1**	**0,52**	**1,61E-02**	***Calcium transport, Response to oxidative stress***
**248843**	T2bp	**0,52**	**1,32E-02**	***Cell Death***
**240393**	*Nfe2*	**0,53**	**1,00E-02**	**DNA replication, Nervous System Development and Function**
**305580**	*Abcc12*	**0,53**	**9,52E-03**	**Cardiovascular Disease, Cellular Development, Embryonic Development**
**253103**	*Adck4*	**0,53**	**9,28E-03**	**Cardiovascular Disease, Cellular Development, Embryonic Development**
**235246**	Cyp2d26	**0,54**	**5,56E-03**	***Detoxification, Clearance of drugs***
**200884**	*Sall3*	**0,54**	**5,56E-03**	**Cancer, Cell growth and proliferation, Respiratory Disease**
**235326**	*Kcnj5*	**0,54**	**4,30E-03**	**Cell Death, Neurological Disease, Nervous System Development and Disease**
**187962**	1700055C04Rik	**0,56**	**2,11E-03**	***Unknown (Reproductive system)***
**310508**	*Arfgef2*	**0,56**	**1,79E-03**	**Cardiovascular Disease, Cellular Development, Embryonic Development**
**271541**	Dusp4	**0,56**	**1,56E-03**	**Carbohydrate metabolism, Lipid metabolism, Small molecule Biochemistry**
**189538**	*Hist2h3c1*	**0,57**	**1,29E-03**	**DNA replication, Nervous System Development and Function**
**192597**	*Grit*	**0,58**	**6,36E-04**	***Neural Development***
**273128**	*Nrbp2*	**0,59**	**2,86E-04**	***Embryonic mouse brain development, Neuronal differentiation***
**259059**	Ralb	**0,62**	**6,20E-05**	**Carbohydrate metabolism, Lipid metabolism, Small molecule Biochemistry**
**194060**	Defb13	**0,65**	**1,47E-05**	***Host's innate defense***
**197262**	***Sprr2g***	**0,67**	**3,88E-06**	**Cell Death, Neurological Disease, Nervous System Development and Disease**
**278391**	1110038D17Rik	**0,79**	**8,61E-10**	***Unknown (Embryonic Development)***
**284995**	GeneBank A530045M11, AI604229, AA174363	**0,81**	**1,62E-10**	**Unknown**

Differentially expressed genes detected by the microarray analysis are listed with the calculated differential ratios (log2) and the Bonferroni *p *values (Bonf Pval). The top functions were deduced either using the Ingenuity pathways analysis software http://www.ingenuity.com or by looking at the expression pattern and putative functions attributed to those genes (italized annotations). Italic names: genes potentially involved in cellular development and differentiation. Bold-faced type name: gene potentially involved in cell death and disorders, including response to oxidative stress. Italic and bold-faced type names: genes potentially involved in both sets of functions.

QPCR was applied to confirm the microarray results for the genes suspected differentially expressed with the observed highest log2 ratios, using the same pools of brain RNAs and to assess the Cre-induced *Prnp *invalidation (Table 5). This latter point was confirmed at both 10 and 14 weeks with a highly significant knockdown of the Tg37-transgene expression in the brain of Tg37-NFH-Cre transgenic mice. The down-regulation observed is less important than that detected in FVB/N *Prnp*^-/- ^mice which is an expected result since the Cre deletion is limited to the neurons, due to the tissue-specificity of the NFH promoter. The slight difference observed between 10 and 14 weeks might suggest that at 10 weeks, the deletion process is not fully complete. The percentage of brain cells that have a deleted Tg37 transgene following Cre activation was previously estimated to be around 29 - 37% [14]. Our data suggest that these cells are among those that express the transgene the most.

**Table 5:** QPCR analysis of the expression of candidate genes resulting from microarray studies comparing Tg37xNFH-Cre versus Tg37 mice.

Genes	Tg37xNFH-Cre	Tg37xNFH-Cre	
	**Microarray**	**QPCR**	**Protein**

	**(log2 ratio)**	**(ΔΔ CT)**	

10 weeks			
*Glrx3*	-0.6	0.34	glutaredoxin 3
*Atp13a1*	0.81	-0.08	ATPase type 13A1
*Prnp Tg37*	ND	-5.62	Prion protein
14 weeks			
*AV451297.1*	-2.62	-0.02	Hypothetical protein
*Ifitm3*	-1.41	-0.12	Interferon-induced transmembrane protein 3
*Erf1*	-1.19	-1.12	Eukaryote class I release factor
*Bace1*	-0.48	-0.02	beta-site APP-cleaving enzyme 1
*BB217622.2*	0.81	0.64	Unknown
*Riken D17*	0.79	0.31	Unknown
*Fgf2*	0.48	0.47	fibroblast growth factor 2
*Prnp Tg37*	ND	-6.43	Prion protein

Differentially expressed genes detected by the microarray analysis are listed with the calculated differential ratio (log2) alongside the observed delta-delta CT resulting from the QPCR experiment. The comparative C_t _method is known as the 2 - ^[delta][delta]Ct ^method, where delta delta C_t _= [delta]C_t, sample _- [delta]C_t, reference_. Age of the analyzed mice is mentioned.

The microarray data were confirmed for 5 out of 9 analyzed genes (Table 5). Among the non-confirmed genes is the above mentioned AV451297.1 putative gene. This gene, located in mouse chromosome 17 and/or mouse chromosome 6, encodes for a hypothetical protein, and its transcription was only reported as an EST in ES cells. The non-confirmation of the differential expression of this gene was surprising since its estimated log ratio was relatively high. Blast alignment of the microarray oligonucleotide corresponding to this gene allowed us to design primers that recognized a family of mouse ESTs that encompass this sequence (Table 6). However, this oligonucleotide aligns with various regions of the mouse genome (data not shown), located on several chromosomes, and we therefore cannot exclude that a transcript, originating from one of these regions, that will not be amplified by our set of primers is responsible for the observed differential expression. Although showing a down-regulated expression in Tg37^+/- ^NFH-Cre^+/- ^mice in both the micro-array analysis and the QPCR experiment, the ratio observed by QPCR was relatively lower than could be expected for the *ifitm3 *gene. The primers used for the QPCR were chosen in order not to amplify the other *ifitm *gene family mRNAs (see Table 6 for the QPCR primer sequences). However, they share some homology with the ankyrin repeat domain 12 (data not shown) which might interfere with the obtained results. The other non-confirmed genes correspond to differentially expressed genes showing very low log2 ratios on the microarray results, between -0.8 and + 0.8. Overall the qPCR obtained data for these genes strengthen the relative transcriptomic stability of the *Prnp *knockout brain. The microarray and QPCR data were consistent for the *Erf1 *transcriptional deregulation (Table 5). We further analyzed the expression level of this gene in the brain of *Prnp*-knockout mice expressing or not the NFH-Cre transgene. No difference was observed (data not shown), demonstrating that the Cre expression does not significantly influence the expression profile of this locus and thus that its observed deregulation in our experiment results from the *Prnp *invalidation. Although we cannot formally exclude that some of the other deregulated genes listed in Tables 3 and 4 results from the Cre expression, this data strongly suggest that the neuronal PrP depletion is responsible for the observed transcriptional modifications.

**Table 6:** List of the used oligonucleotides

FVB/N PrP-/- mice	
	
Name	SEQUENCE (5' -3')
*Prnp 5'*	CAACCGAGCTGAAGCATTCTG
*Prnp 3'*	CGACATCAGTCCACATAGTC
*scg5 5'*	CCTTTATGAGAAAATGAAGGG
*scg5 3'*	GGACAGATTTCTTTGCCACA

**Tg37xNFH-Cre mice**	
**Name**	**SEQUENCE (5' -3')**

*Ifitm3 5'*	TCAGCATCCTGATGGTTGTT
*Ifitm3 3'*	TGTTACACCTGCGTGTAGGG
AV451297.1 5'	CCCGAAGCGTTTACTTTGAA
AV451297.1 3'	CCCTCTTAATCATGGCCTCA
*Erf1 5'*	TCGCTCCACCAACTAAGAAC
*Erf1 3'*	AAACACGGGAAACCTCACC
*Prnp *Tg37 5'	GAAGGAGTCCCAGGCCTATT
*Prnp *Tg37 3'	GCAGGAATGAGACACCACCT
*Glrx3 5'*	CATAAGCATGGTGTCCAAGG
*Glrx3 3'*	TGCCTTCTCTGCTTCGTAGA
*Riken D17 5'*	AAGCCTTCATAGCGAGTGGA
*Riken D17 3'*	TTCCAGACAAGTGGACCTGA
*Bace1 5'*	TCGACCACTCGCTATACACG
*Bace1 3'*	CTCCTTGCAGTCCATCTTGAG
*Fgf2 B 5'*	AGCGGCTCTACTGCAAGAAC
*Fgf2 B 3'*	GCCGTCCATCTTCCTTCATA
*Atp13a1 5'*	CGTGACAAGGGTGAAGATGG
*Atp13a1 3'*	ATAGTAAGAGAAGGCATTCC
*BB217622.2 5'*	CCAGTTCCGTCAAAGTACCC
*BB217622.2 3'*	CATGCAGATCTTCAGGTCCA
*β-actin 5'*	TGTTACCAACTGGGACGACA
*β-actin 3'*	GGGGTGTTGAAGGTCTCAAA

The sequences of the oligonucleotides used in the QPCR experiments are listed; including those of the housekeeping gene that was used in the three described analyses. The sets of primers for the Erf1, Glrx3, BB217622.2 and the AV451297.1 loci were designed within a single exon. All the other sets were designed over exon-exon borders.

The log ratios observed for the other detected differentially expressed genes were relatively low. However, it has to be kept in mind that this invalidation only involved neuronal cells, and probably not all of them [14], and thus that transcriptomic modifications occurring within this cell population will be diluted by the heterogeneous cell composition of the analyzed adult brain tissues. It could also suggest that the biological relevance of the observed variation is doubtful. Only 3 genes were found to be differentially expressed at both stages, *Kcnj5*, *1810030J14Rik *and *Synaptotagmin 11 *(Tables 3 and 4), of which only *1810030J14Rik *was found to behave similarly between these two time-points. This apparent discrepancy could be explained when the function of the differentially express genes was further analyzed, either using the Ingenuity pathways analysis or by looking at the expression pattern and putative functions attributed to those genes (Tables 3 and 4). At 10 weeks, the detected genes appear to reflect a cellular response to an oxidative stress (Table 3), which is in phase with putative physiological functions attributed to PrP [5,6]. Some of the detected genes also suggest that at that stage, the PrP depletion might induce damaged synaptic trafficking and cell death, two cellular pathways into which PrP is also suspected to have a role (Table 3 and [5,6]). At 14 weeks, the differentially expressed genes are rather evocative of a remodeling of the nervous system (Table 4). Most of the identified genes are indeed involved in cellular development and neuronal differentiation. The stage-specific modulation of the *Kcnj5 *and *Synaptotagmin II *are in agreement with this proposed scenario. So although a few genes are found to be differentially expressed with low detected log ratios, the functions of these genes appear relevant and consistent.

Overall, our data suggest that invalidation of the *Prnp *gene does not induce gross modification of the adult mouse brain transcriptome. When this event happens a few days before the analysis is performed, we cannot however exclude that the few moderately differentially expressed genes that are then detected indicate a physiological PrP role in adult neuronal homeostasis, synaptic transmission, survival and differentiation. Several hypotheses might explain this unexpected low responsiveness to the invalidation of such an evolutionary conserved protein at least in mammals. An explanation might be that the cellular response to the lack of PrP does not involve transcriptomic alteration but modifications of post-transcriptomic regulations. This latter suggestion is attractive in regards with the recently published miRNA specific signature observed in mouse-scrapie affected brains [34]. However, it is in contradiction with the lack of detectable modification of the brain proteome of *Prnp*^-/- ^mice [29], which could rather suggest that the variations observed in the miRNA profile is a consequence of the scrapie infection rather than of a PrP loss of function. It is also possible that the overall brain lack of response is due to the fact that the invalidation of the *Prnp *gene only affects a small subset of the brain cellular population and is therefore not detectable in our present transcriptomic analysis or in the proteomic experiment of Crecelius *et al. *[29]. Indeed, if as suggested PrP positively regulates neural precursor proliferation in adult [35], the effect of its invalidation might be difficult to assess without prior purification of this cell type. PrP might also be essential for brain response to specific stressful physiological conditions and that the physiological role of this gene was therefore not challenged in the classical presently used breeding conditions. Another attractive explanation would be that PrP has a key function only during early embryogenesis, as its developmental regulation [7-9] and recently published experimental data involving gene knockdowns [36,37] suggest. Following this early developmental stage, the physiological role of PrP might be less crucial and/or redundant under normal physiological conditions. If so, it would be important to repeat transcriptomic and proteomic analyses at earlier embryonic stages, at the time *Prnp *is turned on or under specific breeding conditions.

## Conclusions

This paper documents the lack of drastic brain transcriptomic modification following the *Prnp *invalidation either at the zygotic stage or in adult neuronal cells of the brain tissue. It is consistent with the recently reported proteomic stability of the brain of such PrP-knockout mice [29] and questions some of the obtained results using *in vitro *cell cultures [25-27]. It might suggest that either this gene knockdown affects the animal physiology at a different developmental stage than the one studied here or that it has to be analyzed in certain particular environmental conditions and/or in more specific cell types.

## Methods

### Mouse brain material and DNA or RNA extraction

Mouse brains from five 6 weeks old female FVB/N and FVB/N *Prnp *^-/- ^animals [10,30] were collected and frozen in liquid nitrogen immediately after decapitation. Homozygous Tg37 mice were crossed with heterozygous NFH-Cre (Cre 22) mice and the genotype of the resulting pups determined by PCR analysis of their tail-extracted genomic DNA as previously described [14]. Sets of two males and two females of Tg37^+/- ^NFH-Cre^+/- ^or of Tg37^+/- ^NFH-Cre^-/- ^genotype, respectively, and of either 10 or 14 weeks old were obtained and their brains collected and frozen in liquid nitrogen immediately after decapitation. All animal manipulations were done according to the recommendations of the French Commission de Génie Génétique.

RNA extractions for the microarray were made using the RNeasy Lipid Tissue Midi kit (Qiagen cat no. 75842). Each brain sample was treated independently. RNA concentration was calculated by electro-spectrophotometry and the RNA integrity checked with the Agilent Bioanalyser (Waldbroom, Germany). Pools were obtained by mixing equal amounts of total RNA from each individual sample.

### Microarray analysis

Microarray analysis was carried out at the Unité de Recherche en Génomique Végétale (URGV, Evry, France) using the mouse 25K array [38] containing 24109 mouse gene-specific oligonucleotides. Amplified RNAs were produced from 2 μg of total RNA from each pool with the "Amino Allyl Message Amp aRNA amplification kit" (Ambion). Five g of amplified RNAs were reversed transcribed with SuperScript II Reverse Transcriptase kit (Invitrogen) in the presence of cy3-dUTP or cy5-dUTP for each slide as previously described [39]. Hybridizations, array scanning and image analyses were performed as previously described [40], using a GenePix 4200A scanner and GenePix Pro 3.0 (Axon Instruments).

The statistical analysis was based on two dye-swap. For each array, the raw data comprised the logarithm of median feature pixel intensity at wavelengths 635 nm (red) and 532 nm (green). No background was subtracted. In the following description, log-ratio refers to the differential expression between the two tissues analyzed: either log2 (red/green) or log2 (green/red), according to the experimental design. An array-by-array normalization was performed to remove systematic biases. First, features that were considered by the experimenter to be badly formed (e.g. because of dust) were excluded (flagged) 100 in the GENEPIX software. Then we performed a global intensity-dependent normalization using the Loess procedure [41] to correct the dye bias. Finally, on each block the log-ratio median was subtracted from each value of the log-ratio of the block to correct a print-tip effect.

To determine differentially expressed genes, we performed a paired t-test. We assumed that the variance of the log-ratios was the same for all genes, by calculating the average of the gene-specific variance. In order to assess this assumption, we excluded spots with a variance too small or too large. Raw P-values were adjusted by the Bonferroni method, which controls the family-wise error rate [42]. A gene is declared differentially expressed if its adjusted P-value is lower than 0.05. The statistical analysis was performed by using the package R anapuce http://www.agroparistech.fr/mia/doku.php?id=productions:logiciels#anapuce1.1.

### QPCR analysis

Three μg of purified RNA was reverse transcribed with SuperScript II Reverse Transcriptase kit (Invitrogen) according to the manufacturer's protocol. Quantitative real-time PCR analysis was performed using ABI PRISM 7000 Sequence Detection System (Applied Biosystems) and SYBR Green (Applied Biosystems). Primers used are listed in Table 6. These primers were designed over exon-exon borders if possible. If not, a RT - control was added in the experiment to control for the absence of DNA contaminant. Normalization was done using the β-actin housekeeping gene. The temperature cycle used comprised 45 cycles at 95°C for 15 sec and 60°C for 1 min. A dissociation curve followed, this was comprised of 95°C for 15 sec, 60°C for 1 min and 95°C for 10 sec. Each sample was analysed in triplicate and data analysed using the Delta-Delta Ct method.

### Scg5 promoter analysis

Genomic DNA was extracted from tail biopsies as previously described [43]. The *Scg5 *proximal promoter was amplified by PCR using the set of primers 5'-CCAGGAATCTCCTAAGATCCTGG-3' and 5'-GACATCCTCTAGATTTTAGAATTACC-3' [33]. The amplified DNA fragment was gel purified and sequenced [44].

## Authors' contributions

SC, RY, and GT performed the RNA purifications and labeling and microarray hybridizations, RY, SLG and GT performed the QPCR experiments. MV, BP and CP bred and obtained the transgenic mice and BP, GT and RY collected the tissue samples used. FB, LST, SB, MLMM and JPR supervised the microarray experiment and performed statistical analysis of the results. VB, FLP, HL and JLV designed and supervised the overall experiment and prepared the manuscript. All authors read and approved the final manuscript.
